# Expert Panel Workshop Consensus Statement on the Role of the Environment in the Development of Autoimmune Disease

**DOI:** 10.3390/ijms150814269

**Published:** 2014-08-15

**Authors:** Christine G. Parks, Frederick W. Miller, Kenneth Michael Pollard, Carlo Selmi, Dori Germolec, Kelly Joyce, Noel R. Rose, Michael C. Humble

**Affiliations:** 1 Epidemiology Branch, National Institute of Environmental Health Sciences (NIEHS), National Institutes of Health (NIH), Research Triangle Park, NC 27709, USA; 2 Environmental Autoimmunity Group, NIEHS, NIH, Bethesda, MD 20892, USA; E-Mail: millerf@mail.nih.gov; 3 Department of Molecular and Experimental Medicine, the Scripps Research Institute, La Jolla, CA 92037, USA; E-Mail: mpollard@scripps.edu; 4 Division of Rheumatology, Allergy and Clinical Immunology, University of California, Davis, CA 95616, USA; E-Mail: carlo.selmi@unimi.it; 5 Rheumatology and Clinical Immunology, Humanitas Clinical and Research Center, Rozzano 20089, Milan, Italy; 6 National Toxicology Program, NIEHS, NIH, Morrisville, NC 27560, USA; E-Mail: germolec@niehs.nih.gov; 7 Department of History and Politics, Drexel University, Philadelphia, PA 19104, USA; E-Mail: kaj68@drexel.edu; 8 John Hopkins Center for Autoimmune Disease Research, Bloomberg School of Public Health, Baltimore, MD 21205, USA; E-Mail: nrose2@jhu.edu; 9 Division of Extramural Research and Training, NIEHS, NIH, Research Triangle Park, NC 27709, USA; E-Mail: humble@niehs.nih.gov

**Keywords:** National Institute of Environmental Health Sciences (U.S.), consensus, autoimmune diseases, mechanisms, environmental exposures, epidemiology, exposure assessment

## Abstract

Autoimmune diseases include 80 or more complex disorders characterized by self-reactive, pathologic immune responses in which genetic susceptibility is largely insufficient to determine disease onset. In September 2010, the National Institute of Environmental Health Sciences (NIEHS) organized an expert panel workshop to evaluate the role of environmental factors in autoimmune diseases, and the state of the science regarding relevant mechanisms, animal models, and human studies. The objective of the workshop was to analyze the existing data to identify conclusions that could be drawn regarding environmental exposures and autoimmunity and to identify critical knowledge gaps and areas of uncertainty for future study. This consensus document summarizes key findings from published workshop monographs on areas in which “confident” and “likely” assessments were made, with recommendations for further research. Transcribed notes and slides were reviewed to synthesize an overview on exposure assessment and questions addressed by interdisciplinary panels. Critical advances in the field of autoimmune disease research have been made in the past decade. Collaborative translational and interdisciplinary research is needed to elucidate the role of environmental factors in autoimmune diseases. A focus on exposure assessment methodology is needed to improve the effectiveness of human studies, and more experimental studies are needed to focus on causal mechanisms underlying observed associations of environmental factors with autoimmune disease in humans.

## 1. Introduction

Autoimmune diseases result from a damaging immune response directed against the body’s own tissues. Of the 80 individual autoimmune diseases, common examples include rheumatoid arthritis (RA), Type 1 Diabetes (T1D), and the autoimmune thyroid diseases; others may be rarer, but as a group afflict 5%–9% of the U.S. population [1,2,3,4,5]. Chronic and incurable, these diseases constitute a major public health problem with high individual suffering and societal costs. The majority of autoimmune diseases disproportionately affect women with a few notable exceptions (e.g., Type 1 diabetes) [5,6]. Some are more common or severe in different racial/ethnic populations (e.g., lupus in African Americans) and age-groups (e.g., Type 1 diabetes in children). The reasons for these differences and underlying cause(s) of autoimmune disorders remain largely unknown, but are likely to involve both genetic and environmental factors [2]. This is well illustrated by the concordance rates observed in monozygotic *versus* dizygotic twins, which differ significantly but remain in most instances are well below 50% [7].

Over the years, a number of trans-NIH committees and NIH-supported workshops have examined the role of the environment in the development of autoimmune disease [8,9,10,11]. In 2003, the NIEHS co-sponsored the “Environmental Factors in Autoimmune Disease” workshop (along with other NIH partners, the United States Environmental Protection Agency (EPA), and the American Autoimmune Related Diseases Assoc., Inc., Eastpointe, MI, USA), and in 2005 co-sponsored the “Workshop on Lupus & the Environment: Disease Development, Progression and Flares” [10,11]. These produced recommendations for research initiatives on the role of the environment in autoimmune disease. One specific recommendation was to facilitate interactions between specialties and encourage multidisciplinary approaches to improve overall knowledge of the hazard, mechanisms, and outcomes associated with specific environmental exposures.

To evaluate the state of the science and provide an opportunity for interactions between specialties, the NIEHS convened an “Expert Panel Workshop to Examine the Role of the Environment in the Development of Autoimmune Disease” on 7–8 September 2010. The goal was to bring together an interdisciplinary group of experts from the environmental health science and autoimmune research communities to review the literature and evaluate the state of the science, recommending productive directions for research on environmentally related autoimmune disease via the publication of a consensus statement.

The workshop utilized a format implemented in previous expert panel meetings [12,13,14]. Participants were selected for three panels examining the role of the environment in the development of autoimmune disease: molecular mechanisms and receptor dynamics; animal models; and epidemiology/human studies. Each panel defined the areas for review and reported their findings, grouped by confidence levels: (1) “Based on existing evidence we are confident of the following…”; and (2) “We consider the following to be likely but require confirmation…”. The panels were asked to identify key knowledge gaps and broad themes for future research. Each group determined the scope of environmental factors they would consider, but all included chemical, physical, biological exposures. A fourth panel was tasked with addressing issues in exposure assessment, a topic of importance to the advancement of human studies.

During the second half of the workshop, four trans-disciplinary panels were formed consisting of members from each of the original review panels. Each panel discussed a common set of over-arching question using the same framework as the initial reviews and reported the findings according to confidence level with summary recommendations for broad themes for future investigations.

In this workshop report and consensus document, we summarize the individual panel reviews on mechanisms, animal models, and human studies, published elsewhere in full [15,16,17]. Because of the volume of literature reviewed, citations here are limited to key publications and examples. This report also summarizes findings from the panel on human exposure assessment and the interdisciplinary panel discussions.

## 2. Workshop Summaries

### 2.1. Mechanisms

The specific mechanisms leading to autoimmune diseases and the effects of environmental exposures on those mechanisms remain largely unknown. A variety of experimental studies are beginning to identify mechanisms by which environmental agents may induce or enable tolerance breakdown and/or autoimmune disease. Focusing on environmental exposure-based autoimmunity, the panel examined six major sub-topics summarized in Table 1 [15] including: effects on innate immunity such as Toll-like receptor (TLR) activation by xenobiotics; adjuvant effects and inflammatory responses; B cell activation; direct effect impairing the immune function, such as T-helper 17 (Th17) cells T regulatory (Treg) cells; and modifications of self-antigens.

**Table 1:** Panel findings on mechanisms involved in the role of environmental factors and development of autoimmune disease.

We Are Confident of the Following	We Consider the Following Likely, but Requiring Confirmation	Broad Themes to Be Pursued in Future Investigations
B cells		
Dysfunctions of B cell tolerance checkpoints are directly correlated with autoimmune disease in murine models; B cells modulate autoimmunity positively and negatively as secretors of antibodies and inflammatory cytokines, as antigen presenting cells to autoreactive T cells, and secretors of anti-inflammatory cytokines such as IL-10; Follicular B cells (B2) are a major source of autoreactive pathogenic antibodies; B cells secreting pathogenic autoantibodies can emerge when somatic hypermutation occurs outside of germinal centers; Sex hormones like estrogen and prolactin can differentially activate autoreactive B cell populations from different subsets (e.g., B2).	B1 cells and marginal zone B cells can modulate autoimmunity by exacerbating it through secretion of autoreactive antibodies and/or by down-modulating it through secretion of anti-inflammatory cytokines; B10 cells appear to exclusively secrete IL-10 may be functionally specialized to carry out a negative regulatory role in inflammation and autoimmunity.	The roles of B1 and marginal zone B cells in autoimmunity; The role of the recently discovered B10 cell population in autoimmunity; The survival/apoptotic pathways that when dysregulated lead to expansion and survival of autoreactive B cells (such as the BAFF/BlyS receptor system and CD40); Tolerance checkpoint mechanisms regulating the formation of high affinity autoreactive B2 cells both in and outside the germinal center; Environmental agents with the potential to disrupt B cell function.
T-helper 17 (TH17) cells		
Dysregulated Th17 cell activity can lead to pathology, as in chronic inflammatory diseases such as asthma or inflammatory bowel disease; Th17 cells are involved in multiple sclerosis (MS), rheumatoid arthritis (RA), Crohn’s disease and psoriasis, where they seem to be involved in disease development and relapse.	Smoking is an important risk factor for RA; and nicotine exerts effects via Th17 cells; Aryl-hydrocarbon Receptor (AhR) binding by aromatic hydrocarbons and non-halogenated polycyclic aromatic hydrocarbons favors differentiation of Th17 cells and can exacerbate autoimmunity.	The involvement of environmental agents and exacerbation of autoimmune disease through Th17 cells; Therapeutic modulation of Th17 cells.
Innate Immunity		
The interaction between xenobiotics and Toll-like receptor (TLR) is a major mechanism involved in the interaction of environmental factors with autoimmunity development; Innate immune activation via TLR predisposes to toxic-induced inflammation; Adjuvants activate both innate and adaptive immunity, inducing release of chemokines and inflammatory cytokines; Immunization must be accompanied by a strong adjuvant, such as complete Freunds adjuvant, including the mycobacterium component. Incomplete Freund adjuvant results in production of antibodies, but without occurrence of autoimmune diseases.	Altered innate immune responses and dysregulated TLR signaling are a key step in triggering autoimmune diseases, as in virus-induced animal models of type I diabetes; TLR activation in macrophages may predispose cells to toxin-induced inflammatory cytokine production; Active infection or microbial products of infection can provide the adjuvant effect necessary for the induction of many autoimmune disorders.	Allergenicity, functional mimicry of environmental contaminants and physical/chemical elements resembling TLR ligands; Dysregulation of the regulatory B cell (IL-10 producing, CD5+ B cells) through modulation of TLR signaling; Molecular motifs of adjuvants and their physiological receptors that are associated with clinical manifestation of autoimmunity; Genomic predisposition to innate immunity dysfunctions.
T-regulatory (Treg) cells		
Quantitative and qualitative Treg changes contribute to a breakdown in tolerance; The AhR ligand dioxin 2,4,7,8-tetrachlorodibenzo- *p*-dioxin (TCDD) induces immunosuppressive T cells expressing specific Treg markers; AhR ligands also affect skewing of the T cell repertoire towards Treg cells indirectly via antigen presenting cells; TCDD induces indoleamine 2,3-dioxygenase (IDO) transcription to skew the T cell repertoire towards FoxP3+ Tregs; Activation of peroxisome proliferator-activated receptor gamma (PPARγ) promotes Treg induction from naïve cells.	Most studies suggest that AhR activation in T cells or in antigen presenting cells may increase Treg production and therefore decrease autoimmunity, but the opposite outcome is also likely and possibly ligand-specific; Context-specific activation of the AhR by specific ligands may result in either increased or decreased Treg activity; Sex hormones play an important role in Treg development and may underlie female predominance of autoimmune diseases.	Specific chemical, infectious, or physical agents capable of modulating Tregs; Environmental modulators of AhR stimulation; Mechanisms of sex-specific Treg changes.
Modification of self-antigens		
The majority of human proteins undergo post-tranlational modification (PTM) and these modifications or lack thereof may lead to tolerance breakdown; PTM may explain the tissue specificity of autoimmune diseases; MS pathogenesis includes PTM that increase the complexity of myelin proteins through the autoimmune response or neurodegenerative processes; In RA, citrullination is an apoptotic PTM that seems to be helpful in opening protein conformation and favoring cleavage processes; In PBC, cholangiocytes do not covalently link glutathione to lysine-lipoyl groups during apoptosis leading to accumulation and exposure to potentially self-reactive antigens, accounting for bile duct specific pathology.	Multiple self-protein modifications (phosphorylation, glycosylation, acetylation, deamidation) can lead to either T or B cell responses to self-antigens; Serum autoantibodies to modified self antigens may bind either modified or unmodified forms and thus be crucial to effector immune reaction in target tissues; Mercury-induced cell death results in formation of a unique and more immunogenic 19 kDa cleavage fragment of fibrillarin.	Mechanisms by which citrullination and glutathionylation lead to tolerance breakdown in susceptible individuals; The role of glucosylation in MS and other autoimmune diseases; Experimental models to prove that autoantigens can be modified to increase their immunogenicity; Technologies to reverse or induce PTM in animal models of autoimmunity.
Modification of DNA methylation		
DNA methylation profiles are associated with environmental factors including prenatal tobacco smoke, alcohol, and environmental pollutants; The importance of DNA methylation in regulating immune function is suggested by two rare congenital diseases, Silver-Russel and Beckwith-Wiedemann syndromes; Changes in DNA methylation in specific peripheral immune cell types are associated with autoimmune diseases.	Phenotypic differences are increased with age in twins in a trend coined as “epigenetic drift”, due to different environmental exposures, and may explain late-onset autoimmunity; Specific impairments in epigenetic regulation in immune cells may be responsible for immune-tolerance breakdown through hypo-methylation of genes or involvement of transcription repressors; Recent genome-wide association studies demonstrate that genomics significantly predispose to systemic lupus erythematosus (SLE) onset, but experimental studies indicate that epigenetic mechanisms, especially impaired T and B cell DNA methylation, may be one of these factors.	The functional effects *in vivo* of DNA methylation changes under different environmental and genomic conditions; The development of new therapeutic molecules capable of preventing or counteracting DNA methylation changes in a cell-specific manner; The DNA methylation changes in the target cells and not only in the rapidly accessible effector immune cells.

#### 2.1.1. Effects on Innate Immunity

Two major related mechanisms within innate immunity were discussed: Toll-like receptor (TLR) activation, and the role of adjuvants. The Toll-like receptors (TLRs) are pattern recognition receptors that play a key role in the effectiveness and function of the innate immune system. The panel was confident that TLR activation and signaling is a major mechanism linking environmental factors to development of autoimmunity. TLR deficiency impacts both disease severity and autoantibody profiles in pristane-induced autoimmunity [18]. Moreover, TLR-related pathways are likely to play a role in virally-induced animal models of autoimmune disease (e.g., Type 1 diabetes), and active infections or microbial exposures may provide the necessary adjuvant effect for the induction of many autoimmune diseases. Recommendations for further research included investigation of environmental factors and TLR activation, and TLR-related effects on regulatory B cells [19,20,21].

Adjuvants are agents that non-specifically stimulate the immune system without direct antigenic effects, including TLR-mediated effects on innate immune response and factors that modulate the adaptive immune response. The panel concluded that adjuvants (e.g., complete Freund’s) are important in the development of autoimmune disease. Further research is needed to characterize molecular aspects of adjuvants and receptors involved in autoimmune diseases, and on genetic risk factors that may modify autoimmune responses to environmental adjuvants and triggers of the innate immune response [11,22,23].

#### 2.1.2. B Cell Activation

One of the two major cell types in the adaptive immune response, B cells secrete pathogenic auto-antibodies and can also present antigens to auto-reactive T cells. A breakdown in central tolerance (in the bone marrow) is a major contributor to autoimmunity in many experimental models. Determining the contributions of B cell subtypes in autoimmune disease and the role of environmental factors in biasing their activation is critical. The panel reported a high degree of confidence in the role of follicular B cells and the influence of sex hormones (e.g., estrogens) via this mechanism, and that research is needed to identify effects of environmental exposures on B cell development and function, e.g., environmental estrogens [24,25,26,27].

#### 2.1.3. T-Helper 17 Cells

T-helper 17 (Th17) cells, an interleukin 17 (IL17)-producing subset of T-helper cells, play an important role in the adaptive immune response and mechanisms leading to autoimmunity and chronic inflammation. The panel was confident that dysregulation of Th17 cells contributes to chronic inflammatory pathology, and that Th17 cells are involved in development and relapse of several autoimmune diseases [28,29].

Increasing evidence suggests that xenobiotics, allergens and micronutrients can influence Th17 cells at multiple levels. For example, smoking, a risk factor for RA and other autoimmune diseases, exerts effects on Th17 cells through nicotine exposure. Aromatic hydrocarbons and non-halogenated polycyclic aromatic hydrocarbons also induce differentiation of Th17 cells through binding at the Aryl hydrocarbon Receptor (AhR), exacerbating autoimmunity. Given the important role of Th17 cells on development and exacerbation of autoimmunity, more research is needed on the effects of environmental exposures on Th17 cells [30,31,32].

#### 2.1.4. T Regulatory (Treg) Cells

T regulatory (Treg) cells play a key role in the maintenance of immune tolerance, and can dampen or suppress activation of the immune system. Experimental studies demonstrate a number of mechanisms through which environmental agents may affect Treg induction or function. The panel was confident that changes in Treg cells play a role in loss of tolerance for self-antigens. Strong evidence supports 2,4,7,8-tetrachlorodibenzo-*p*-dioxin (TCDD)-induction of suppressive T cells with Treg markers, along with mechanisms involving antigen-presentation, through which AhR ligands skew the T cell repertoire towards Treg production [33,34,35]. The peroxisome proliferator-activated receptor gamma (PPARγ) receptor is activated by a wide variety of environmental chemicals (e.g., phthalate esters), promoting Treg induction [36,37].

Confirmation is needed that the context-specific activation of the AhR by specific ligands may result in either increased or decreased Treg activity. Sex-hormones are likely to regulate Treg development, and may underlie the female predominance of most autoimmune diseases. The panel concluded that studies should focus on environmental factors capable of modulating Treg and AhR activity and also consider the role of chemical mixtures and direct stressors, such as ultraviolet (UV)-light [35,38,39,40].

#### 2.1.5. Modification of Self-Antigens

Post-translational modification (PTM) is the chemical modification of a protein following its synthesis, e.g., methylation, phosphorylation, acetylation, lipidation, or glycosylation, occurring on 50% to 90% of proteins in the human body. An environmental exposure may alter PTM, affecting immunogenicity of self-proteins and triggering an autoimmune response. PTM may explain tissue specificity of some autoimmune diseases. For example, PTM increases complexity of myelin proteins through autoimmune or neurodegenerative processes in MS. Conversely, lack of PTM during apoptosis alters protein degradation and leads to accumulation of self-reactive antigens related to bile duct specific pathology of primary biliary cirrhosis (PBC) [41,42].

Confirmation is needed that multiple types of environmentally-induced PTM may lead to B and T cell reactivity to self-antigens, and that self-reactive antibodies react to both modified and unmodified forms of antigen. One example is the binding of mercury to fibrillarin to create a modified self-antigen and the generation of a new cleavage fragment due to proteolysis following mercury-induced cell death. Research is also needed on mechanisms by which citrullination (e.g., linking smoking to RA) leads to loss of self-tolerance and studies of PTM biomarkers across the natural history of autoimmune diseases [43,44,45,46,47].

#### 2.1.6. Modifications of DNA Methylation

The field of epigenetics examines the regulation of the genome through modifying mechanisms not involving changes in the nucleotide sequence itself, such as DNA methylation and histone acetylation. Environmental factors can affect epigenetic gene regulation, and so understanding the role of epigenetic modifications in the development of autoimmunity is an important topic for future study. The panel confidently noted the association of DNA methylation profiles with environmental exposures, including prenatal tobacco smoke, alcohol use, and environmental pollutants (*i.e.*, particulate matter).

Confirmation is needed that ageing-related phenotypic changes arise due to exposures over the lifetime, contributing to development of autoimmune diseases later in life. Studies are needed to show whether loss of tolerance is related to specific exposure-associated impairments in regulation of epigenetic processes and confirming impaired methylation of B and T cell DNA in relation to systemic lupus erythematosus (SLE) risk. Recommendations include research on the *in vivo* effects of DNA methylation under different environmental conditions and target tissue differences in DNA methylation associated with autoimmune diseases [7,48].

### 2.2. Animal Models

Animal models have been used extensively in the study of autoimmune disease and the role of environmental exposures. The panel focused their attention on studies of non-therapeutic chemical, biological, and physical factors associated with autoimmune outcomes as summarized in Table 2 [17]. A high level of confidence was reached if multiple studies from different laboratories confirmed the same findings. For findings considered likely and requiring further confirmation, there needed to be significant support, including multiple studies from a single laboratory, or repetition of some but not all findings in multiple laboratories.

The panel noted that autoimmune responses to chemical factors are species and strain-specific. Autoimmune animal models (predominately rats and mice) are typically genetically manipulated or inbred strains that spontaneously develop disease or autoimmunity induced by immunization with specific antigens. Some studies of environmental factors in autoimmunity involve the *induction* of autoimmune diseases or autoimmunity in non-susceptible, inbred strains. Due to the genetic complexity of disease susceptibility, autoimmune effects may not be observed. Thus, studies may also investigate environmental effects on models of spontaneous autoimmune disease, in which case the effects of exposure may include *exacerbation* or acceleration of disease expression. Because of the great depth of the literature in this area, the published review on animal models and autoimmune diseases was limited in scope [17]; an additional white paper on the full workshop review session is available by request.

Studies provided conclusive evidence that forms of inorganic mercury (HgCl_2_, vapor, amalgam) can induce systemic autoimmune disease in rats (transient) and mice [49], and exacerbate or accelerate systemic disease in lupus-prone mice [50]. Several mineral oil components and other hydrocarbons can induce inflammatory arthritis in rats [51]; and one component, 2,5,10,14-tetramethylpentadecane (TMPD or pristine) induces lupus-like disease and inflammatory arthritis in some strains of mice [52]. With a high degree of confidence, the panel also noted a role for specific pathogens (*i.e.*, Streptococcal group A, Coxsackie B virus) and exacerbation of autoimmune thyroiditis by iodine in genetically predisposed animal models.

**Table 2:** Panel findings studies of animal models in the role of environmental factors and development of autoimmune disease.

We Are Confident of the Following	We Consider the Following Likely, but Requiring Confirmation	Broad Themes to Be Pursued in Future Investigations
Forms of inorganic mercury (HgCl_2_, vapor, amalgam) induce systemic autoimmune disease in rats (transient) and mice, and exacerbates systemic autoimmune disease in lupus-prone mice; Several mineral oil components and certain other hydrocarbons can induced an acute inflammatory arthritis in some rat strains; The mineral oil component 2,6,10,14-tetramethylpentadecane (TMPD or pristane) induces lupus-like disease and inflammatory arthritis in several strains of mice; For a limited number of pathogens there is a clear association with development of autoimmune diseases; Excess iodine increases the incidence of autoimmune thyroiditis in genetically predisposed animal models.	Gold causes (transient) nephropathy in rats. Gold and silver cause autoimmune responses, but not autoimmune disease, in mice; but the ability of silver and gold to exacerbate spontaneous autoimmune disease requires study; Silica exacerbates autoimmune disease but more studies are needed using more species/strains and a wider range of doses and exposure routes; Trichloroethylene (TCE) exacerbates systemic autoimmunity although responses are often limited and transient. Studies of autoimmune liver disease are needed with additional species/strains and in developmental studies; TCDD exposure during fetal or early neonatal development may promote autoimmunity; Organochlorine pesticides may enhance lupus-like disease in a predisposed mouse strain; Sunlight/ultraviolet (UV) light exposure exacerbates lupus in genetically prone mice.	Studies should be “shaped by what is observed in humans, not by what is possible in mice” [53]; Studies should not be restricted to a “gold standard” animal model. Multiple models should be investigated to reflect human genetic heterogeneity; When using spontaneous disease models it is important to consider whether environmental exposures directly impacts idiopathic autoimmunity, or reflects environmental factor-specific autoimmunity; More studies on the effects of environmental factor exposure on expression of autoimmunity during different stages of life (gestational to adulthood) are needed.

The panel considered a wide range of other associations to be likely, but needing confirmation. Examples include some heavy metals (gold and silver), though more studies are needed of other metals (organic mercury, cadmium, lead, and arsenic) to confirm observed effects. Silica exacerbates autoimmune disease in lupus models, but studies are needed in different species/strains and across a wider range of exposure routes and doses. Evidence is suggestive that trichloroethylene (TCE) can exacerbate systemic autoimmunity in a limited or transient manner, and UV radiation/sunlight is likely to exacerbate lupus in genetically prone mice. Developmental exposures (fetal/neonatal) to TCDD may promote systemic autoimmunity, supporting the idea that early exposures may influence the developing immune system and subsequent development of disease. Findings that organochlorine pesticides (e.g., dichlorodiphenyltrichloroethane [DDT]) enhance autoimmune disease in a susceptible mouse model also require confirmation.

The panel noted several themes for future use of animal models to study environmental autoimmunity and disease. Above all, it was recommended that findings in animal models should not be the only driving force for human studies, which should be “shaped by what is observed in humans, not by what is possible in mice”. A single mouse strain cannot encompass the genetic heterogeneity in human populations, so studies should not be limited to “gold standard” animal models. Rather, the effects of environmental exposures should be tested on multiple models, and if necessary, humanized models. Exposure effects should be examined during all stages of life, from gestation to adulthood. When using spontaneous autoimmune models, studies should consider whether exposures exacerbate or accelerate idiopathic autoimmunity or induce more specific “environmentally-associated” forms of autoimmunity. The panel also recommended specific improvements to animal studies, including use of disease markers from easily obtained biological fluids (e.g., blood) to enhance comparisons with human studies.

### 2.3. Epidemiology/Human Studies

Findings of the epidemiology/human studies review are summarized in Table 3 [16]. The panel restricted their focus to peer-reviewed studies published in the last 30 years using defined Medline searches of the primary literature. Meta-analyses were examined with respect to study identification, inclusion and exclusion criteria, and the methods used to abstract and derive summary estimates. When the design and analysis methodology was deemed acceptable, the study estimate was used to summarize evidence through the period covered by that review; additional studies published subsequent to the meta-analysis were also reviewed.

Diseases of focus included: Crohn’s disease (CD), gluten-sensitive enteropathy (GSE, celiac disease), Graves’ disease (GD), Hashimoto’s thyroiditis (HT), idiopathic inflammatory myopathies (IIM), multiple sclerosis (MS), primary biliary cirrhosis (PBC), rheumatoid arthritis (RA), systemic lupus erythematosus (SLE), systemic sclerosis (SSc), type 1 diabetes (T1D), and ulcerative colitis (UC). Additional diseases were examined if a substantial literature existed for a particular exposure e.g., eosinophilia myalgia syndrome [54]. Exposures were grouped in three broad classes: chemicals, physical factors, and biologic agents.

**Table 3:** Panel findings on human studies on the role of environmental factors and development of autoimmune disease.

We Are Confident of the Following	We Consider the Following Likely, but Requiring Confirmation	Broad Themes to Be Pursued in Future Investigations
Chemicals		
Crystalline silica (quartz) contributes to development of several systemic autoimmune diseases, including RA, systemic sclerosis (SSc), SLE and anti-neutrophil cytoplasmic antibody (ANCA)-related vasculitis. Solvents contribute to development SSc. Smoking contributes to development of anti-citrullinated protein antibody (ACPA)-positive and anti-rheumatoid factor. (RF)-positive RA (with an interaction with the shared eptiope genetic susceptibility factor).	Solvents contribute to development of MS. Smoking contributes to development of seronegative RA, MS, SLE, Hashimoto’s thyroiditis (HT), Graves’ disease (GD) and Crohn’s disease (CD). Current smoking protects against development of ulcerative colitis (UC).	There is insufficient evidence on the role of metals, including those associated with animal models of autoimmunity, e.g., mercury. The identification of single causal agents within groups of exposures is needed (e.g., specific solvents or pesticides contributing to increased risk for the group). Studies are needed on plasticizers (e.g., phthalates and bisphenol A), some of which may be endocrine or immune disruptors, and have been associated with other immune mediated diseases. There is insufficient evidence on the role of cosmetics in autoimmune diseases.
Physical factors		
An inverse association exists between increased ultraviolet radiation exposure and risk of developing MS.	Ionizing radiation contributes to development of HT and GD.	There is insufficient evidence on a possible protective role of ultraviolet radiation on type 1 diabetes (T1D). Prospective data are needed on sun exposure as a risk factor for SLE (prior to early clinical symptoms) and dermatomyositis.
Biologic agents		
Ingestion of gluten contributes to development of gluten-sensitive enteropathy (GSE). Ingestion of certain lots of l-Tryptophan contributes to development of eosinophilia myalgia syndrome. Dietary intake of 1,2-di-oleyl ester (DEPAP)- and oleic anilide-contaminated rapeseed oil contributes to development of toxic oil syndrome.	Epstein-Barr virus (EBV) infection contributes to MS development. Early introduction of complex foods contributes to development of T1D and GSE. Low dietary vitamin D intake and blood levels contribute to development of MS.	Studies are needed on MS and vitamin D in racial/ethnic groups with darker skin (associated with UV-associated vitamin D deficiency), and examining dose-effects. Prospective data are needed on vitamin D and other autoimmune diseases. Additional studies are needed on associations of food chemicals, dyes, or additives. Prospective studies are needed on nitrates/nitrosamines and T1D.

A “confident” association was based on evidence from multiple studies in different populations using different designs; robust evidence of an overall association (*i.e.*, high magnitude risks or based on high quality or established exposure assessment methods); a dose-response relationship, or effect differences by disease subtype or genetic factors supporting biologic plausibility. “Likely” associations were based on similar body of research, but missing key evidence, such as a temporal relationship, less consistent results, or fewer studies.

#### 2.3.1. Chemical Factors

The panel was confident that crystalline silica exposure contributes to development of several systemic autoimmune diseases, including RA, SSc, SLE, and anti-neutrophil-cytoplasmic antibody (ANCA)-associated vasculitis [55,56]. Evidence also supports an association of solvent exposure (in general) and development of SSc [57]. The panel was confident that smoking contributes to anti-citrullinated peptide antibody (ANCA) and anti-rheumatoid factor (RF)-positive RA, interacting with the shared epitope genetic risk factor [58,59].

Evidence also suggests smoking is likely to play a role in development of seronegative RA, MS, SLE, HT, GD, and CD, while protecting against UC [60,61]. Research needs varied by disease type; findings are inconsistent on the relation of smoking with MS and SLE. General solvent exposure may also contribute to development of MS, but more research is needed using improved exposure assessment methods. Major research gaps include studies of metals associated with autoimmunity in animal models and identification of specific causal agents within general groups of exposures (e.g., pesticides or solvents). Based on their endocrine and immune-disrupting qualities, the panel recommended studies of plasticizers in development of autoimmune diseases.

#### 2.3.2. Physical Factors

The panel was confident in the inverse association of higher UV exposure and risk of MS [62]. Ionizing radiation is likely to contribute to development of autoimmune thyroid diseases (HT and GD), though several limitations contribute to a degree of uncertainty. These include studies of medical radiation therapy that did not distinguish HT from general hypothyroidism, and inconsistency in findings from nuclear testing fallout and accidental radiation contamination. Research gaps include the role of UV exposure as a protective factor against T1D and as a risk factor for dermatomyositis and SLE, with prospectively collected data and attention given to the period prior to potential clinical symptoms related to sun sensitivity.

#### 2.3.3. Biological Factors

The panel was confident about the role of gluten in the development of GSE [63]. Strong evidence supports a role of specific environmental exposures in eosinophilia myalgia syndrome [54] and toxic oil syndrome [64].

It was also deemed likely that Epstein-Barr virus (EBV) infection contributes to development of MS, and that early introduction of complex (e.g., solid) foods contributes to development of T1D and GSE. The panel noted that MS may be associated with lower vitamin D intake and blood levels, but research was recommended in non-Caucasian populations at higher risk of UV-related vitamin D deficiency, and studies of dose-effects. Prospective studies on vitamin D and other autoimmune diseases were also recommended. A research gap was noted on the role of food additives, dyes, and chemicals, and prospective studies on the role of nitrates and nitrosamines in T1D.

Broad recommendations for future epidemiologic research included studies of multiple exposures and chemical mixtures, reflecting the real life complexity of human exposures. More studies are needed on exposure-related risks within specific disease phenotypes and in the context of genetic risk factors, such as the association of smoking with RA in the context of anti-citrullinated peptide antibody positivity and the shared epitope. Research needs also include defining critical windows in the timing of exposures and latencies relative to developmental stage, understanding dose-response relationships, and identifying mechanisms.

### 2.4. Exposure Assessment in Human Studies

Addressing a unique need recognized by the epidemiologic and clinical research community, the workshop also included a focus group on exposure assessment, bringing together experts in epidemiologic methods and exposure measurement technologies. Most autoimmune diseases are chronic and the relevant timing of exposures is not well established. Because most of the diseases are individually rare, retrospective case-control studies are often the most efficient design, with assessment methods based on questionnaires, relying on self-report and recall. The accuracy of internal dose estimates may be improved using exposure biomarkers, such as serum pesticides or metals; however, many exposures (e.g., silica) do not have easily accessible biomarkers, and for others (e.g., pesticides or metals), current biomarker levels may misclassify exposures during relevant time-periods of disease initiation or progression.

The ability to identify environmental risk factors for autoimmune diseases depends heavily on the availability of rigorous exposure assessment methods that can be applied in different populations and allow comparisons across studies. Standardized questionnaires, such as those offered by the PhenX Toolkit [65], may address this need for some exposures, such as smoking. But studies identifying or confirming risk factors for autoimmune diseases often require greater detail on specific agents, such as solvents or pesticides, which vary by disease. Assessment methods may also need to be tailored to specific populations or settings, such as the methods used to assess occupational silica exposure in women in rural *versus* urban settings [66,67]. A life-course perspective is also critical, given the general lack of knowledge on the relevant time-windows of exposures in human studies and the well-established influence of developmental exposures on the immune system.

New technologies in exposure measurement are being developed with potential applications in autoimmune disease research. Personal measurement technologies may have limited usefulness in assessing exposures years or decades prior to disease onset, but may help validate questionnaires on current or recent exposures or in long-term follow-up studies. Studies are also using new technologies to link geographic exposures with autoimmune disease studies, for example: air pollution and RA [68], UV radiation and dermatomyositis [69].

The panel recommended an integrated approach to improve exposure assessment in human autoimmune disease research. While adequate tools may exist to assess some relevant exposures (e.g., smoking, silica), they are not widely accessible to researchers and their use typically requires collaboration with experts in assessment methodology. The application of new technologies may improve the accuracy and efficiency of existing methods, for example, using measurement data to validate questionnaires. Analytic methods that utilize complex data resources to model past exposures [70,71,72], or simultaneously take into account multiple exposures (e.g., Exposure-wide association studies; EWAS, [73,74]), will be important resources for future studies. Applications of information technologies are also needed to create useful databases incorporating biomarkers, questionnaires, measurement studies, and data analysis guidelines for autoimmune researchers. These investments require a focus on the “big picture” and integration across disciplines, with research and infrastructure development that requires support and cooperation across multiple agencies conducting public health and scientific research, such as the Centers for Disease Control and Prevention, National Institutes of Health, Environmental Protection Agency and National Science Foundation.

Specific recommendations for advancing exposure assessment in environmental autoimmune diseases research include: (1) improving sensitivity, specificity, and dose estimates for established risk factors for one or more specific autoimmune diseases (*i.e.*, silica, solvents, UV radiation); (2) a focus on disease risk in high exposure groups (occupational, military, and other risk populations); (3) consideration of highly prevalent or emerging “new” exposures (e.g., obesity, phthalates); and (4) prospective exposure assessment in susceptible populations (e.g., family members autoimmune disease patients, women). Resources are also needed to guide clinicians in the collection and interpretation of environmental exposure data [75]. Information collected in clinical settings is often limited to smoking and current occupation. In order to target specific exposures (e.g., silica or solvents), clinically applicable questionnaires are need to integrate across a wide variety of industries and occupations, including past as well as ongoing exposures.

### 2.5. Transdisciplinary Breakout Panels

Integrated responses from the four transdisciplinary breakout panels identified a range of specific needs and opportunities advancing research in environmental autoimmunity.

#### Topic 1—Do animal models recapitulate disease observed in humans following exposure?

In the majority of examples animal models do not entirely mimic human autoimmune diseases. Across the range of 80+ autoimmune diseases, most have complex etiologies, while animal models are designed to minimize complexity to foster an understanding of mechanisms. Exceptions in which animal models recapitulate features associated with environmental autoimmunity/disease in humans include pristane-induced lupus, toxic oil syndrome, Coxsackie virus-induced myocarditis, and l-tryptophan-associated eosinophilia–myalgia syndrome (EMS). The (NZBxNZW)F1 model also recapitulates human SLE (with central nervous system (CNS) involvement, vasculitis, dermatitis), and models of ultraviolet B (UVB) exposure and SLE exacerbation correspond well with the common clinical perception of flares and lupus. In these cases, however, the complexity of the animal model approaches that of the human disease, making a mechanistic understanding more difficult to achieve. A number of models support human data on environmental risk factors for autoimmune diseases, but need further development and characterization, including models of TCE-induced autoimmunity designed to investigate the relationship of solvents and autoimmune diseases, including SSc.

In several instances, animal models of environmentally induced autoimmune need to be promoted when there are good epidemiological data supporting an association. A prime example is that of silica and silicate exposures, which have been associated with multiple autoimmune diseases in humans. These findings are corroborated by relatively few studies in animal models, and so more work is recommended, particularly for the inhalation route of exposure. Other examples include studies of smoking effects on MS, and early dietary exposures and diabetes. (At the same time, epidemiological studies should also be promoted when good mechanistic data exists based on animal studies (such as mercury-induced SLE-like disease, and effects of mercury and other heavy metals associated with exacerbations)). Panel discussions highlighted a need for genetically diverse animal models of autoimmunity to reflect the heterogeneity of human populations and for studies of gene-environment interactions. Models are also needed that reflect relevant doses and exposure mixtures, and that mimic the sex differences often seen in human autoimmunity.

#### Topic 2—Do exposures associated with autoimmune disease *in vitro* and in animal models have relevance to exposures in human populations?

Although exposure levels in human studies of autoimmunity are often unclear, animal models generally use higher doses to shorten experimental periods or reveal underlying mechanisms as proof-of-principal. Studies of risk factors associated with human autoimmune disease may provide hints as to what exposure types and doses to evaluate in animal models. There are reasonably good *in vitro* data for some exposures (e.g., mercury) where the exposures achieved following animal dosing *in vivo* may be comparable to levels found in humans [76]. Other relevant associations seen in experimental studies may include EBV associated with MS, mineral oil components with RA/SLE/inflammatory arthritis.

Further research was recommended to explore similarities and differences between animal models and humans in the metabolism, pharmacokinetics, distribution/internal dose, and target organ dose of specific xenobiotics related to autoimmunity. Given the current limitations of exposure assessment in human studies, efforts are needed to determine the validity of biomarkers of exposure, for example methylation arrays and self-protein reactivity arrays to citrullinated or glycosylated proteins.

#### Topic 3—How do susceptible populations, time frames of exposure, or genetic predisposition contribute to exposure-related autoimmune disease? 

Many intrinsic factors (e.g., genetics, gender, age) work together in complex ways to contribute to the development of exposure-related autoimmune disease; these interactions are likely to be both complex within given exposure/disease relationships and variable across different types of exposures and diseases. Animal models provide examples of differences in autoimmune susceptibility by age or genetic background (e.g., mercury [77]). Human studies also provide proof of principle—for example the difference in relationship between smoking, autoantibodies and RA phenotypes depending on the human leukocyte antigen (HLA)-DR4. Animal models suggest the time frame of exposure is likely to be important, for example prenatal/early life TCDD exposures may have different outcomes than exposures more proximal to autoimmune onset [78,79]. Confirmation is needed in humans that exposure timing (e.g., early life exposures *versus* later life exposures) affects disease risk, which may be difficult given the long latency time and challenges in assessing early life exposures. Other examples that timing or genetic factors are likely to be important include the timing of the introduction of complex foods during infancy and T1D, and UV exposure interacting with genetic risk factors in MS risk [16].

The interactions of age, gender, genetics and windows/timeframes with environmental exposure(s), and the impact of their relative contributions, are critical to a more complete understanding of the development of autoimmune diseases. Recommendations for further investigation included: mechanisms underlying sex or gender differences in autoimmune-effects of solvents and other environmental exposures, and timing of exposures and role of genetic susceptibility for several exposures, including sunlight (e.g., protection against MS), vitamin D, silica, and EBV infection. Findings on genetic susceptibility in human autoimmune diseases, e.g., HLA-DR4 and RA, should be incorporated into animal models (e.g., humanized mice) when possible.

#### Topic 4—To what extent does ability to quantify environmental exposures limit our ability to identify factors associated with human autoimmune disease?

Limitations in methods and technologies to assess environmental exposures in humans can substantially hinder the detection of exposures related to the development of autoimmune disease. Better exposure data would produce more accurate dose-response estimates and lead to identification of more risk factors. For example, using a less sensitive and specific method for silica exposure made a substantial difference in observed association with lupus [66]. Because autoimmune diseases in humans are relatively uncommon, case-control studies are often needed to achieve sufficient numbers of cases for analyses. Thus, there is a particular need for the development and dissemination of methods to assess historical exposures.

The low incidence of many of the individual diseases (especially in men) presents a logistical challenge for studies in exposure-enriched populations, e.g., occupational cohorts, which often have higher levels of specific exposures and sometimes also include measurement data. Likewise the rarity of some exposures (e.g., high level silica exposure) present challenges in studies conducted in the general population or patient registries.

There is an urgent need to develop cost-effective non-invasive methods to quantify those environmental exposures most likely related to autoimmune disease in both human populations and animal models. New technologies must be developed, and emerging technologies exploited. Recommendations were provided in the context of the panel discussion specifically devoted to the topic of exposure assessment (above).

#### Topic 5—How well do mechanistic studies *in vivo* or *in vitro* relate to clinical outcomes?

There is a paucity of studies that relate autoimmune disease mechanisms with clinical measures and exposure-associated autoimmune diseases. It was generally agreed that experimental/mechanistic studies are focused on models used for proof of principle, and so findings do not correspond well with what is known in humans. Table 4 reviews knowledge on mechanisms related to three exposures identified with the greatest confidence as being associated with human disease in this workshop: smoking, silica, and solvents.

One of the most solid examples is the mechanistic relationship between citrullination, a form of post-translational modification and the development of autoantibodies to citrullinated proteins, thought to suggest a causal role for smoking in the development of RA [80]. Experimental data is somewhat limited, however studies of gene-environment interactions and other human studies are providing additional clues to etiologic pathways [81,82,83]. The role of other smoking-related mechanisms in RA including heat shock gene expression and related autoantibodies is less clear [84,85]. Notably, animal models suggest nicotine-associated delays in development of arthritis [86,87].

Despite the strong and consistent associations of silica with multiple systemic autoimmune diseases, there relatively little evidence on the possible mechanisms underlying this relationship. Silica exposure can exacerbate lupus in rodent models [88]; though the mechanisms by which this occurs are not established, hypotheses include an adjuvant effect. Other evidence coming from human studies includes associations of silica exposure with dysregulation of apoptosis and balances of T-helper/Treg [89,90], and associations of silica disease-specific autoantibodies in a highly exposed population [91].

Lastly, although the solvent/SSc association seen in human studies is consistent, it lacks support from specific animal models of SSc. However, indirect evidence comes from the observed specificity of the solvent association in patients with disease-specific autoantibodies [92]. By contrast, a large body of literature provides evidence of one specific solvent, Trichloroethylene, in immune disease regulation, such as increased IFN-γ and decreased IL-4, and disease aggravation/acceleration in lupus models [93].

**Table 4:** Evidence and hypothesized mechanisms underlying autoimmune disease associations with smoking, silica, and solvents.

Exposure-Disease Association in Humans	Evidence on *in Vitro* and *in Vivo* Mechanisms
Smoking and seropositive-RA	Post-translational modification—antigen citrullination and anti-cyclic citrullinated peptides (CCP) antibodies [80,82];
	Nicotine and Th17 activation [86,87];
	Upregulation of heat shock gene expression [84] *;
	Disease relevant autoantibodies (RF, anti-HSP70) [85] *.
Silica and RA/SLE/SSc/ANCA-vasculitis	Aggravation of lupus in animal models [88];
	Adjuvant effect-apoptotic debris [88];
	Dysregulation of apoptosis [90] *;
	Disease relevant autoantibodies (anti-dsDNA, anti-Ro/SSA, anti-La/SSB antibodies in silica associated SLE) [91] *;
	Altered CD4+/CD4+ CD25+ T cell ratio [89] *.
Solvents and SSc	Accelerated autoimmunity in animal models [93]
	SSc disease relevant autoantibodies (anti-Scl-70) [92]
	Increased IFN-γ, reduced IL-4 [93] *

* Similar observations made in animal studies.

Patterns of T and B cell skewing, antibody and cytokine profiling may be useful biomarkers of autoimmune disease or predisposition, and represent mechanisms linking the environment with disease initiation. Data in human studies on autoantibody profiles in patients, controls and high-risk populations would allow examine exposure-associations with specific autoantibodies and whether exposures impact development or progression of disease susceptible populations (e.g., due to genetic or autoantibody profiles). Many models have been established to study mechanisms regulating autoimmune diseases, and these need to be tested in terms of their role in exposure-mediated disease.

Inclusion of more mechanistic endpoints in human autoimmune studies will entail important logistical adjustments. The collection of patient samples must provide access to live cells for *in vitro* investigations, e.g., preserving peripheral mononuclear blood cells rather than merely serum samples for future functional assays and phenotyping. This will require greater collaboration and understanding between basic environmental health scientists, autoimmune clinicians, and environmental epidemiologists.

#### Topic 6—How well do *in vitro* mechanisms relate to *in vivo* mechanisms in animals or effects of human exposures?

There is a growing list of examples (e.g., Hg and silica) showing concordance between mechanistic findings from *in vitro* and *in vivo* studies in laboratory animals. The types of mechanisms for which there is agreement include: apoptosis, co-stimulation, antigen clearance and presentation, cytokines and signaling. In most model systems there is good correlation between *in vitro* and *in vivo* outcomes, for example AhR modulation [94,95]. At the same time, *in vitro* systems are often too limited to recapitulate observations from animal models.

At the same time, there is inadequate data on whether most exposure-related autoimmune mechanisms are found in humans at relevant exposure levels, though there are some suggestive data regarding cytokines, lymphocyte subsets, DNA methylation and other epigenetic factors, e.g., for silica and air pollution [90,96,97,98]. Oxidative stress, specific environmental chemical receptors, and environmentally-induced TLR activation likely play a role in development of human disease [20,99]. While *in vitro* evidence suggests AhR ligands can affect T cell differentiation [34,100,101], additional animal *in vivo* and human studies are needed to determine the importance of these findings. Analyses of different molecular and biological outcomes following AhR activation with different classes of ligands (e.g., dioxins, PCBs, dietary flavonoids) should also be pursued as a model for context-specific environmental chemical signaling.

## 3. Summary and Conclusions

### 3.1. Overall Advances in this Field

Critical advances in the field of autoimmune disease research include a growing understanding of the contribution of antigen specific T cell subsets, B cell antibody repertoire, and antigen presentation. Specific to the role of environmental factors in autoimmune disease etiology, there has been an improved understanding of the role of specific signaling molecules (e.g., TLRs, AhR). Other advances include the emergence of new technologies for assessing molecular markers (e.g., gene, methylation, and antibody arrays), genetic manipulations in animal models to define mechanisms and potential use of the GWAS (genome-wide association studies) repository. A key finding from human studies includes the identification an environmental exposure (cigarette smoke), which interacts with genetic factors to promote specific RA phenotypes and for which there are relevant mechanistic data, which provides a model for future studies of environmental autoimmunity integrating exposure, genotype, and phenotype.

### 3.2. Conclusions and Recommendations

More “translational” epidemiological studies of environmental autoimmunity are needed and should be guided by mechanisms defined in model systems and *vice versa*. An integrated, multidisciplinary approach is critical, and programs should be established to provide opportunities for collaboration and improve communication between epidemiologists, exposure scientists, and basic cellular/molecular biologists, *i.e.*, fostering of interdisciplinary research through forums, funding and training. Funding opportunities need to be specifically targeted towards autoimmunity and environmental factors. Better coordination across the diverse disciplines and agencies conducting autoimmune research may help to encourage collaborations. Such coordinated efforts may also promote a more cohesive body of knowledge through studies of multiple autoimmune diseases with similar underlying mechanisms, and shared genetic or environmental risk factors.

An important need for human autoimmune research is the availability of high-quality, validated measurement tools. Similar to efforts to characterize the genome, new technologies should be harnessed to address the critical need to characterize human environmental exposures. An environment-wide association (*i.e.*, “exposome”) database linked to common questionnaires would facilitate epidemiological studies. More data are also needed on the contribution of psychosocial factors, infections, complex mixtures and susceptibility factors to the development of autoimmune diseases. Biomarkers identified by mechanistic studies should be applied to epidemiologic research in the context of relevant exposure measures. Investments in high quality exposure measures and biological markers will increase the ability to identify environmental contributions to the etiopathogenesis of autoimmune diseases.

Finally, a consensus-based approach should be developed to define autoimmune phenotypes (rather than diseases), which may improve comparability between human studies and animal models. The focus on studying diseases defined by classification criteria may limit interpretation of animal model data and the ability to identify human exposure cohorts using the broadest disease definitions. Conversely, there is a need for animal models to better represent phenotypes that occur in human diseases (e.g., CNS-lupus). Some environmental exposures may cause diseases characterized by a mixture of outcomes or multiple phenotypes that do not fit standard diagnostic criteria. Outbreak investigations should collect data to characterize the emerging phenotypes, and include the preservation and archiving of biological specimens. Long-term follow-up of affected individuals is critical to assess phenotypes that might develop with long latency.
